# Psychometric properties and local normative references of PSC-17, RCADS-25, CATS-2, SNAP-IV, MCHAT-R/F, and CAST: data from a nationwide sample in Greece

**DOI:** 10.1186/s41687-026-01032-1

**Published:** 2026-03-11

**Authors:** André Simioni, Julia Luiza Schafer, Lauro Estivalete Marchionatti, Kenneth Schuster, Caio Borba Casella, Katerina Papanikolaou, Efstathia Kapsimalli, Panagiota Balikou, Giorgos Gerostergios, Kalliopi Triantafyllou, Maria Basta, Nikos Zilikis, Lilian Athanasopoulou, Vaios Dafoulis, Aspasia Serdari, Rafael V. S. Bastos, Peter Szatmari, Ioanna Giannopoulou, Anastasia Koumoula, Giovanni Abrahão Salum, Konstantinos Kotsis

**Affiliations:** 1 Child and Adolescent Mental Health Initiative (CAMHI), Stavros Niarchos Foundation (SNF) & Child Mind Institute (CMI), Athens, Greece; 2 Child Mind Institute (CMI), New York, NY USA; 3 Department of Child Psychiatry, Agia Sophia Children’s Hospital, National and Kapodistrian University of Athens, Athens, Greece; 4 National and Kapodistrian University of Athens, Athens, Greece; 5 Neapolis University Pafos, Pafos, Cyprus; 6 Department of Psychiatry, University Hospital of Heraklion, Crete, Greece; 7 Department of Child and Adolescent Psychiatry, Medical School, Democritus University of Thrace, Alexandroupolis, Greece; 8 Department of Psychology, São Francisco University, Campinas, SP Brazil; 9 Cundill Centre for Child and Youth Depression, Centre for Addiction and Mental Health, Toronto, ON Canada; 10 2nd Department of Psychiatry, Attikon University Hospital, National and Kapodistrian University, Athens, Greece; 11 Department of Psychiatry, University of Ioannina, P.O. Box 1186, Ioannina, 455 10 Greece

**Keywords:** Screening, Mental health, Pediatric, Psychiatry, Child, Adolescent

## Abstract

**Background:**

Health professionals in Greece face barriers in assessing child and adolescent mental health conditions due to the lack of instruments with evidence of validity in local samples. This study addresses this gap by evaluating the psychometric properties and establishing common norms for six globally recognized mental health tools in Greece: the Child and Adolescent Trauma Screen-2 (CATS-2), Pediatric Symptoms Checklist-17 (PSC-17), Revised Children’s Anxiety and Depression Scale-25 (RCADS-25), Swanson, Nolan, and Pelham Scale (SNAP-IV), Modified Checklist for Autism in Toddlers-Revised (MCHAT-R/F), and Child Autism Spectrum Test (CAST).

**Methodology:**

We drew on a nationwide Greek survey comprising 1,756 caregivers and 1,201 children and adolescents (age groups: 1 to 18 years). Using Item Response Theory, we assessed internal consistency and factor models according to Consensus-based Standards for the Selection of Health Measurement Instruments’ (COSMIN) criteria for unidimensionality, local independence, monotonicity, and global model fit. Normative references were calculated using standardized metrics recommended by the Patient-Reported Outcomes Measurement Information System (PROMIS).

**Results:**

Final sample sizes ranged from 1,356 (PSC-17, caregiver version) to 198 (CATS-2, caregiver version). Internal consistency was rated as good to excellent across all scales. Factor analyses supported all scales except the ones assessing autism spectrum disorder: MCHAT-R/F (failing monotonicity) and CAST (failing monotonicity and unidimensionality). Local normative references were usually consistent with international samples.

**Conclusion:**

This toolkit provides essential evidence-based resources for child and adolescent mental health in Greece, offering a scalable model for other underserved settings. Further research with national probabilistic samples is recommended to enhance risk stratification accuracy.

**Supplementary Information:**

The online version contains supplementary material available at 10.1186/s41687-026-01032-1.

## Background

In Greece, significant hurdles for the provision of child and adolescent mental health care still persist. In the aftermath of an economic crisis, there is a shortage and unequal distribution of specialists working in the public sector as well as regional gaps in provision of care [1]. Adding on to that, referral systems and gatekeeping mechanisms are yet to be established, resulting in long waiting times in many child and adolescent mental health services. Within this context of constrained resources, it is of paramount importance to equip health professionals with tools to screen for and stratify the risk of mental health conditions in primary and pediatric care settings. Therefore, interventions and referrals could be oriented by evidence-based assessment of symptom severity [2].

Integrating psychosocial screening tools into pediatric care improves the identification and management of behavioral and emotional conditions, with several guidelines recommending the adoption of standardized core outcome measures [3–5]. In Greece, a recent systematic review highlighted that many widely-used instruments assessing child and adolescent mental health outcomes were either unavailable or lacked validation with local samples, including the Pediatric Symptom Checklist (PSC) and the Swanson, Noland, and Pelham (SNAP-IV) [6]. There was also a shortage of instruments for assessing child abuse and autism spectrum disorders, with key tools such as the Child and Adolescent Trauma Screen-2 (CATS-2), the Child Autism Spectrum Test (CAST), and the Modified Checklist for Autism in Toddlers - Revised (MCHAT-R/F) notably absent [7, 9, 10, 32]. The absence of such tools significantly hinders best practices and research in Greece. In the case of autism spectrum disorders, it has prevented the implementation of systematic screening within the healthcare system and restricted prevalence estimates to administrative diagnostic data [11–13].

The aim of this paper is to contribute to the efforts of the [Child and Adolescent Mental Health Initiative (CAMHI)] in delivering evidence-based resources to enhance child and adolescent mental health care capacity in Greece. As part of this effort, we previously conducted a nationwide survey covering several patient-relevant outcomes, which employed a set of assessment instruments selected for their brevity, availability, and reliability [38]. In the present study, we report the psychometric validation and local normative references of the following instruments: the Child and Adolescent Trauma Screen-2 (CATS-2), Child Autism Spectrum Test (CAST), Modified Checklist for Autism in Toddlers-Revised (MCHAT-R/F), Pediatric Symptoms Checklist-17 (PSC-17), Revised Children’s Anxiety and Depression Scale-25 (RCADS-25), and Swanson, Nolan, and Pelham Scale (SNAP-IV).

## Methods

This study evaluated the measurement performance of the Greek version of six screening instruments (see Table 1), adopting prespecified psychometric criteria for structural validity, reliability, and convergent/discriminant validity and generating local normative references. For each tool, symptom severity bands are presented with a dimensional approach (minimal, mild, moderate, and severe), providing user-friendly, comparable metrics aligned with standards defined by the Patient-Reported Outcomes Measurement Information System (PROMIS) group [14, 15]. Importantly, these bands are interpretive conventions rather than clinically calibrated parameters or diagnostic thresholds. We followed the STrengthening the Reporting of OBservational studies in Epidemiology (STROBE) guidelines (see Supplementary Table 1) [16]. The statistical codes, outcomes, and survey dataset are openly available on our Open Science Framework page at https://osf.io/crz6h/ [17].

**Table 1:** Instruments’ description, factor models, and proposed cutoffs

Domain	Instrument	Description	Informants in our sample¹	Factor model Subscales	Literature proposed cut-off	Reference for Greek adaptation	Distribution
General emotional and behavior screening	Pediatric Symptoms Checklist - Shortened Version (PSC-17) [18, 19]	A 17-item measure assessing psychosocial functioning Rated on a 0-to-2 Likert-scale	Caregiver Self-report	Three-factor model [19, 20]: • Externalizing (7 items) • Internalizing (5 items) • Attention (5 items)	Attention subscale ≥ 7 Internalizing subscale ≥ 5 Externalizing subscale ≥ 7 Total score ≥ 15 [19, 21, 22]	[23]	Distributor’s webpage [24]
Anxiety and depression	Revised Children’s Anxiety and Depression Scale - Short Version (RCADS-25) [25, 26]	A 25-item scale that measures levels of low mood and anxiety. Rated on a 0-to-3 Likert-scale	Self-rated Caregiver-rated	Two-factor model [25, 26]: • Anxiety (15 items) • Depression (10 items)	T-score < 65: low severity T-score 65 to 70: medium severity (borderline clinical threshold) T-score > 70: high severity (above clinical threshold) [25, 26]	[27, 28]	Distributor’s webpage [29]
Attention/hyperactivity deficit disorder	Swanson, Nolan and Pelham Scale (SNAP-IV) [30]	A 26-item instrument for hyperactivity, inattention, impulsivity, and oppositional behavior Rated on a 0-to-3 Likert-scale	Caregiver-rated	Four-factor model adapted from [30]: • Inattention (9 items) • Hyperactivity (5 items) • Impulsivity (4 items) • Oppositionality (8 items)	Oppositional subscale: [25, 29, 31] • < 8 points: Symptoms not clinically significant • 8 to 13 points: Mild symptoms • 14 to 18 points: Moderate symptoms • 19–24 points: Severe symptoms Inattention and hyperactivity/impulsivity^2^ subscales: • < 13 points: Not clinically significant • 13 to 17 points: Mild symptoms • 18 to 22 points: Moderate symptoms • 23 to 27 points: Severe symptoms	[23]	’CAMHI’s Open Science Framework databases Open Science Framework database [17]
Autism spectrum disorder	Modified Checklist for Autism in Toddlers - Revised (MCHAT-R/F) [7, 32]	A 20-item tool assessing neurodevelopment, social interaction, and repetitive behavior in toddlers Yes/no questions	Caregiver-rated	Unidimensional (20 items) [7, 32]	0 to 2 points: low risk 3 to 4 points: cutoffs for risk 8 to 20 points: high risk [7, 32]	[33]	Distributor’s webpage [34]
Autism spectrum disorder	Child Autism Spectrum Test (CAST) [10, 35]	A 28-item^4^ questionnaire on social and behavioral symptoms Yes/no questions rating 1 for each positive answer.	Caregiver-rated	Two-factor model with 28 items [36] • Social contact issues (16 items) • Inflexible and repetitive behaviors (12 items)	≥ 15 points:^3^ possible autism spectrum disorder or social-communication difficulties [10, 35]	[37]	Distributor’s webpage [37]
Trauma	Child and Adolescent Trauma Screen-2 (CATS-2) - symptom Sect. ^5^ [9]	20-item^5^ subscale assessing symptoms of Posttraumatic Stress Disorder (PTSD) Rated on a 0-to-3 Likert-scale	Self-rated Caregiver	Unidimensional (20 items) [9]	≥ 21 points: positive screening for PTSD ≥ 25 points: probable PTSD [9]	[23]	CAMHI's Open Science Framework database [17]

Abbreviations: CAMHI (Child and Adolescent Mental Health Initiative). Notes: ^1^See Koumoula et. al (2024) [38]. ^2^In our model, we separated hyperactivity/impulsivity into two subscales. ^3^This cut-off score refers to the original model; we are using the model proposed by Sun et al. (2014) [36], with cutoff scores still to be established. ^4^While the original instrument comprises 37 items, we used the 28-item version validated in a Chinese sample, which demonstrated the most robust model fit indices. ^5^CATS-2 is preceded by a 15-item yes–no checklist assessing exposure to traumatic events, which was not included in this study

### Participants

**Fig. 1:**
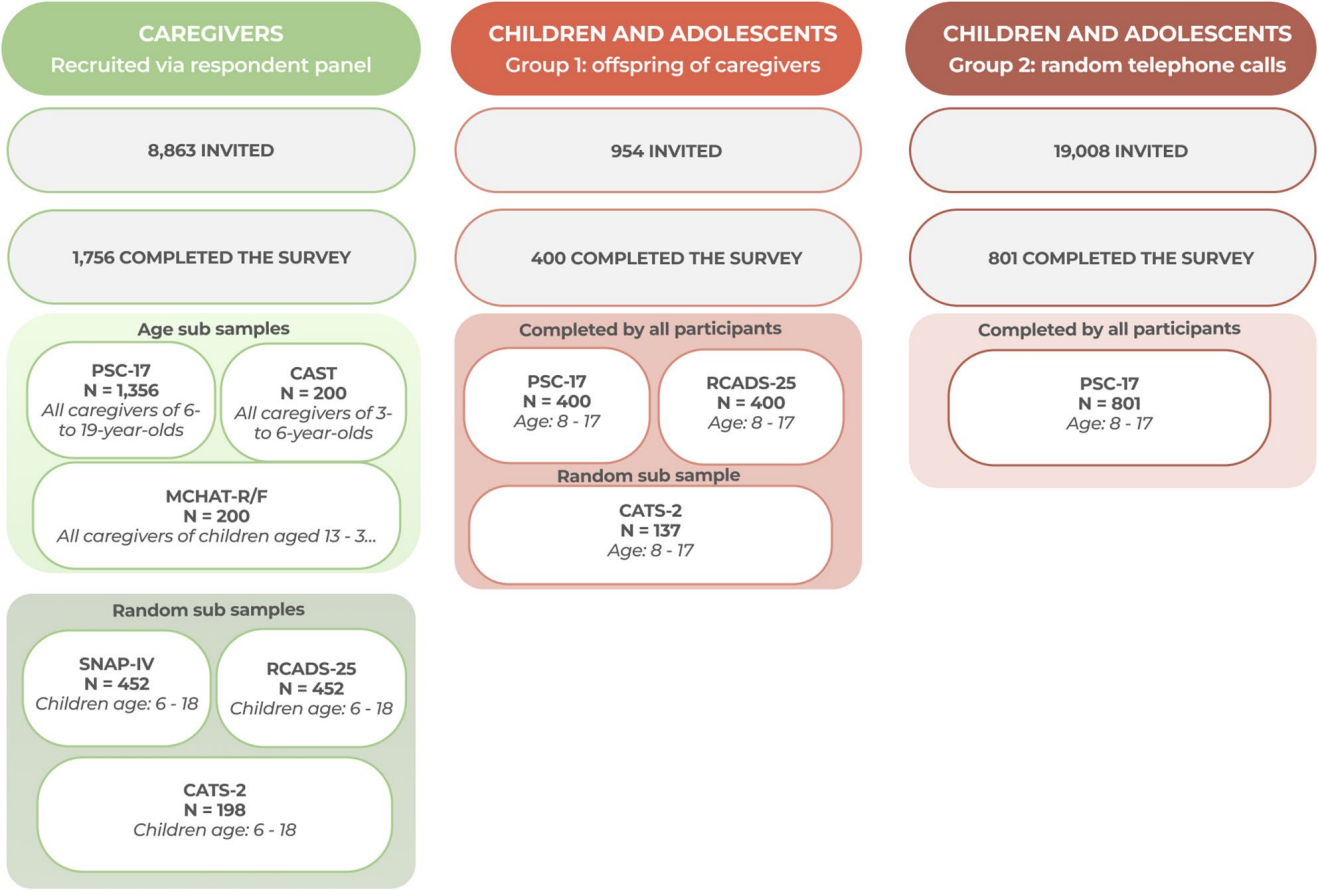
Sampling procedure. Abbreviations: CAST (Child Autism Spectrum Test), CATS-2 (Child and Adolescent Trauma Screen-2), MCHAT-R/F (Modified Checklist for Autism in Toddlers - Revised), PSC-17 (Pediatric Symptoms Checklist − 17 items), RCADS-25 (Revised Children’s Anxiety and Depression Scale − 25 items), SNAP-IV (Swanson, Nolan and Pelham Scale)

We drew on data from a nationwide cross-sectional survey conducted in 2022–2023, including 1,201 children and adolescents (aged 8–17 years) and 1,756 caregivers of children aged 1–18 years (methods detailed in Koumoula et al. (2024) [38]). Instruments were administered to subsamples by age and target group, with additional allocation by random assignment (see Fig. 1).

Caregivers were recruited through a proprietary online respondent panel based on a census sampling frame. An algorithm applied regional residency, offspring sex, and age quotas; 8,863 individuals profiled as parents or legal guardians of children younger than 18 years were invited, and 1,756 participated. One child/adolescent subgroup comprised offspring of participating caregivers: 954 were invited to complete an online questionnaire including adolescent self-report measures paired with caregiver ratings, yielding 400 respondents. A second subgroup of children/adolescents (*n* = 801) was recruited via 19,008 random telephone calls, using census quotas for region and sex, and completed measures of general screening and sensitive outcomes (including substance use and self-harm).

Non-response and refusal were the primary reasons for non-participation. Partial completions were treated as dropouts (28 caregivers; 38 children/adolescents recruited through caregivers; 28 children/adolescents recruited by telephone). Questionnaires were completed electronically, and no item-level missingness was observed across scales.

Data collection was managed with the KoboToolbox [39]. Informed consent and assent were respectively obtained from caregivers and youth participants. Data was collected and preserved according to the General Data Protection Regulation (GDPR) National Policy [40]. The Research Ethics Committee of the Democritus University of Thrace approved the survey [approval number: ∆ΠΘ/ΕΗ∆Ε/42772/307].

### Selection of instruments

Table 1 outlines the instruments used in the nationwide survey [38]. First, we consulted the International Consortium for Health Outcomes Measurement (ICHOM) on patient-relevant outcomes for child and adolescent mental health [41]. We then reviewed the literature on instruments assessing general and specific symptoms of prevalent mental health conditions. We selected tools based on their brevity, availability, and reliability, as recommended by the Consensus-based Standards for the Selection of Health Measurement Instruments (COSMIN) [42]. Three of the selected instruments (PSC-17, SNAP-IV, and CATS-2) had either not been previously translated into Greek or were not freely available in Greek language. In such cases, we performed a structured procedure for cross-cultural adaptation comprising back-and-forth translation by independent translators, synthesis of versions, expert committee appraisal, and pilot testing with the targeted population [43]; each step has been thoughtfully documented in a previous publication [23].

We employed the brief versions of the Pediatric Symptom Checklist (PSC-17) and the Revised Children’s Anxiety and Depression Scale (RCADS-25) [18, 21, 25]. Although the nationwide survey initially used the full-length versions, only the items pertaining to the shortened versions were included in the analysis [38]. For the CATS-2, we validated only the 20-item scale for symptom assessment, which is applied after screening with any positive answer on a 15-item checklist of traumatic events [9].

### Statistical analysis

An Item Response Theory (IRT) approach was conducted to test factor models, internal reliability, and to generate common metrics for each subscale or unidimensional instrument. For normative reference per age group and gender, we converted scores into percentiles, Z-scores, and T-scores, establishing a color-coded classification of symptom severity (minimal, mild, moderate, and severe) [14, 15]. Analysis was performed using the software R version 4.4.3 and the packages *lavaan*, *semTools*, *ltm*, *psych*, and *mirt* [44–49].

#### Selection of factor models

Table 1 shows the factor models tested through Confirmatory Factor Analysis (CFA). Factorial structures were consulted at developers’ distribution pages and supporting literature. For CAST, CATS-2, and SNAP-IV, more than one structure was compared to identify the best fit for our data.

As no developer-recommended structure was available for CAST, we compared models proposed in samples from Spain, Brazil, and China [36, 50, 51]. The Chinese two-factor model (“Sociability/Communication” and “Inflexible/Repetitive behaviors”) was selected for its superior fit to our data and alignment to DSM-5 criteria [36].

There are several structures for CATS-2 based on different diagnostic criteria [9]. While a hierarchical model demonstrated the best performance, there are factors with one or few items that may lead to model over rejection, and its clinical utility is limited due to complex scoring. We have chosen an unidimensional model following DSM-5 structure for its practical utility and superior fit to our data.

While the original SNAP-IV suggests a three-factor model, our data demonstrated a significantly better fit when the ‘hyperactivity/impulsivity’ factor was split into two distinct factors [30]. This four-factor structure (inattention, hyperactivity, impulsivity, and opposition) has been supported in the literature and was selected for this analysis [52].

#### Psychometric analysis

We conducted a factor analysis for each subscale or unidimensional tool, evaluating both item-level and model-level fit [48, 53, 54]. Model fit was assessed using the M2 command from the Mirt package, with estimations of the Root Mean Square Error of Approximation (RMSEA), the Standardized Root Mean-square Residual (SRMR), the Comparative Fit Index (CFI), and the Tucker-Lewis Index (TLI) [48, 54–56]. Item performance was analyzed through factor loadings, residual correlations, item scalability, monotonicity violations, fit indices, and discriminative power. Reliability was estimated using Cronbach’s alpha (α) and McDonald’s omega (ωₜ) [57–59].

#### Evaluation Criteria for Measurement Properties

Criteria were primarily based on the COSMIN manuals and supplemented with additional guidelines as recommended [42, 60, 61]. IRT literature prioritizes a combination of parameters to assess goodness-of-fit at item and test levels as opposed to isolated measures [54, 56, 62, 63].

Internal consistency was considered positive if Cronbach’s alpha (α) or McDonald’s omega (ωₜ) exceeded 0.7 [42, 57, 64, 65]. Values above 0.8 and 0.9 respectively indicated good and excellent reliability.

Factor analysis was deemed positive if the following criteria were met: (1) unidimensionality (at least two of the following: RMSEA ≤ 0.06, SRMR ≤ 0.08, and TLI/CFI ≥ 0.95; RMSEA ≤ 0.08 is also considered acceptable [61]); (2) local independence (residual correlations among items < 0.2 OR the third quartile of correlations < 0.37); (3) monotonicity (item scalability > 0.3 OR adequate looking graphs); and (4) global model fit (unidimensionality criteria attended AND item infit mean squares ≥ 0.5 and ≤ 1.5).

Additionally, factor loadings above 0.3 were considered positive, with values higher than 0.5 classified as very positive [66, 67]. For monotonicity, we also evaluated the number of violations per item. The absence of violations indicated adequate monotonicity, and violations with critical values between 40 and 90 were also considered acceptable [53, 68].

#### Local normative references

Graded Response Models (GRMs) and Graded Rating Scale Models (GRSMs) were employed for parameterization, assigning an IRT score to each participant [55, 69, 70]. Scores were categorized by age group and gender, with T-scores calculated from factor scores (a T-score of 50 represents the reference population mean, and a 10-point difference corresponds to one standard deviation). Symptom level bands followed PROMIS recommendations for standardized metrics, consisting of four ranges: minimal symptoms (T-score < 55), mild symptoms (T-score 55–59), moderate symptoms (T-score 60–69), and severe symptoms (T-score ≥ 70) [15, 71, 72]. Scores were then rescaled according to the range of each T-score-based severity category, with values truncated within each band. Finally, crude scores were linked to IRT-based scores by grouping factor, T-scores, Z-scores, and percentiles for each summed score value.

## Results

Table 2 presents the sociodemographic characteristics of caregivers and children/adolescents who completed each instrument. The PSC-17 (proxy-report, school-age children) had the largest sample size, including all 1,356 caregivers of 6- to 18-year-olds participating in the survey. Some instruments were administered to subsamples, with participant numbers ranging from 452 (RCADS-25, caregiver-rated; SNAP-IV, caregiver-rated) to 137 (CATS-2, self-report).

**Table 2:** Sociodemographic characteristics of participants

Rater	Caregiver version*							Self-report version*		
Instrument	PSC-17 (age 6–18)	PSC-17 (under 6)	RCADS-25	SNAP-IV	MCHAT-*R*/F	CAST	CATS-2	PSC-17	RCADS-25	CATS-2
**Sample size (N)**	*N* = 1,356	*N* = 200	*N* = 452	*N* = 452	*N* = 200	*N* = 200	*N* = 198	*N* = 1201	*N* = 400	*N* = 137
**Children and adolescents: sociodemographic characteristics**										
**Age** *Mean (Min-Max)*	12.1 (6.0-17.9)	4.39 (3.00-5.80)	12.2 (6.0-17.9)	12.2 (6.0-17.9)	2.15 (1.10–2.90)	4.39 (3.00-5.80)	12.1 (6.0-17.9)	12.48 (8.00–17.00)	13.12 (8.00–17.00)	12.96 (8.00–17.00)
**Age group** *N (%)*										
6–9 years	416 (31%)	200 (100%)	143 (32%)	143 (32%)	200 (100%)	200 (100%)	62 (31%)	232 (19%)	60 (15%)	19 (14%)
10–13 years	415 (31%)	0 (0%)	119 (26%)	119 (26%)	0 (0%)	0 (0%)	63 (32%)	477 (40%)	139 (35%)	52 (38%)
14–18 years	525 (39%)	0 (0%)	190 (42%)	190 (42%)	0 (0%)	0 (0%)	73 (37%)	492 (41%)	201 (50%)	66 (48%)
**Gender** *N (%)*										
Female	636 (47%)	96 (48%)	206 (46%)	206 (46%)	100 (50%)	96 (48%)	100 (51%)	589 (49%)	177 (44%)	61 (45%)
Male	719 (53%)	104 (52%)	246 (54%)	246 (54%)	100 (50%)	104 (52%)	98 (49%)	611 (51%)	222 (56%)	76 (55%)
Other	1 (0.07%)	0	0	0	0	0	0	1 (< 0.1%)	1 (0.3%)	0 (0%)
**Health Region** *N (%)*										
Attica	607 (45%)	96 (48%)	193 (43%)	193 (43%)	94 (47%)	96 (48%)	98 (49%)	484 (40%)	181 (45%)	64 (47%)
Piraeus & Aegean Island	52 (3.8%)	7 (3.5%)	15 (3.3%)	15 (3.3%)	9 (4.5%)	7 (3.5%)	11 (5.6%)	50 (4.2%)	15 (3.8%)	5 (3.6%)
Macedonia	278 (21%)	38 (19%)	109 (24%)	109 (24%)	44 (22%)	38 (19%)	37 (19%)	229 (19%)	90 (23%)	31 (23%)
Macedonia and Thrace	125 (9.2%)	12 (6.0%)	42 (9.3%)	42 (9.3%)	10 (5.0%)	12 (6.0%)	12 (6.1%)	95 (7.9%)	33 (8.3%)	9 (6.6%)
Thessaly and Central Greece	109 (8.0%)	19 (9.5%)	40 (8.8%)	40 (8.8%)	12 (6.0%)	19 (9.5%)	18 (9.1%)	120 (10%)	29 (7.3%)	14 (10%)
Peloponnese, Ionian islands, Epirus and Western Greece	131 (9.7%)	20 (10%)	32 (7.1%)	32 (7.1%)	20 (10%)	20 (10%)	12 (6.1%)	160 (13%)	39 (9.8%)	9 (6.6%)
Crete	54 (4.0%)	8 (4.0%)	21 (4.6%)	21 (4.6%)	11 (5.5%)	8 (4.0%)	10 (5.1%)	63 (5.2%)	13 (3.3%)	5 (3.6%)
**Caregivers: sociodemographic characteristics**										
**Age** *Mean (Min-Max)*	43 (19–73)	37.2 (20–58)	43 (22–65)	43 (22–65)	35 (20–62)	37.2 (20–58)	42 (22–60)	Not applicable for self-report questionnaires		
**Gender** *N (%)*										
Female	712 (53%)	122 (61%)	237 (52%)	237 (52%)	122 (61%)	122 (61%)	119 (60%)	Not applicable for self-report questionnaires		
Male	644 (47%)	78 (39%)	215 (48%)	215 (48%)	78 (39%)	78 (39%)	79 (40%)			
**Relationship to the kid** *N (%)*										
Biological parent	1,305 (96%)	192 (96%)	435 (96%)	435 (96%)	193 (97%)	192 (96%)	186 (94%)	Not applicable for self-report questionnaires		
Adoptive parent	21 (1.5%)	2 (1.0%)	5 (1.1%)	5 (1.1%)	3 (1.5%)	2 (1.0%)	8 (4.0%)			
Foster parent	4 (0.3%)	1 (0.5%)	2 (0.4%)	2 (0.4%)	3 (1.5%)	1 (0.5%)	0 (0%)			
Grandparent	2 (0.1%)	2 (1.0%)	1 (0.2%)	1 (0.2%)	0 (0%)	2 (1.0%)	1 (0.5%)			
Aunt/uncle	9 (0.7%)	1 (0.5%)	3 (0.7%)	3 (0.7%)	1 (0.5%)	1 (0.5%)	1 (0.5%)			
Brother/sister	12 (0.9%)	1 (0.5%)	5 (1.1%)	5 (1.1%)	0 (0%)	1 (0.5%)	2 (1.0%)			
Other	3 (0.2%)	1 (0.5%)	1 (0.2%)	1 (0.2%)	0 (0%)	1 (0.5%)	0 (0%)			
**Degree of education** *N (%)*										
Elementary school	5 (0.4%)	1 (0.5%)	3 (0.7%)	3 (0.7%)	0 (0%)	1 (0.5%)	0 (0%)	Not applicable for self-report questionnaires		
Middle school	22 (1.6%)	1 (0.5%)	9 (2.0%)	9 (2.0%)	3 (1.5%)	1 (0.5%)	4 (2.0%)			
High school	198 (15%)	34 (17%)	69 (15%)	69 (15%)	17 (8.5%)	34 (17%)	21 (11%)			
Vocational Lyceum (EPAL)	113 (8.3%)	13 (6.5%)	37 (8.2%)	37 (8.2%)	15 (7.5%)	13 (6.5%)	22 (11%)			
Associate degree / post-lyceum education**	176 (13%)	36 (18%)	47 (10%)	47 (10%)	17 (8.5%)	36 (18%)	26 (13%)			
College	49 (3.6%)	3 (1.5%)	14 (3.1%)	14 (3.1%)	4 (2.0%)	3 (1.5%)	8 (4.0%)			
Technological Educational Institute	219 (16%)	23 (12%)	78 (17%)	78 (17%)	36 (18%)	23 (12%)	35 (18%)			
University	343 (25%)	42 (21%)	122 (27%)	122 (27%)	54 (27%)	42 (21%)	44 (22%)			
Master’s degree	198 (15%)	45 (23%)	62 (14%)	62 (14%)	51 (26%)	45 (23%)	36 (18%)			
Doctorate	29 (2.1%)	2 (1.0%)	9 (2.0%)	9 (2.0%)	2 (1.0%)	2 (1.0%)	2 (1.0%)			
**Employment status** *N (%)*										
Full-time	1,040 (77%)	121 (61%)	355 (79%)	355 (79%)	142 (71%)	121 (61%)	140 (71%)	Not applicable for self-report questionnaires		
Part-time	121 (8.9%)	29 (15%)	38 (8.4%)	38 (8.4%)	15 (7.5%)	29 (15%)	16 (8.1%)			
Unemployment	152 (11%)	45 (23%)	47 (10%)	47 (10%)	32 (16%)	45 (23%)	34 (17%)			
Retired	13 (1.0%)	2 (1.0%)	4 (0.9%)	4 (0.9%)	1 (0.5%)	2 (1.0%)	3 (1.5%)			
Student	30 (2.2%)	3 (1.5%)	8 (1.8%)	8 (1.8%)	10 (5.0%)	3 (1.5%)	5 (2.5%)			
**Family monthly income** *N (%)*										
€500 euros or less	68 (5.0%)	15 (7.5%)	19 (4.2%)	19 (4.2%)	12 (6.0%)	15 (7.5%)	16 (8.1%)	Not applicable for self-report questionnaires		
€501 to €1,000	297 (22%)	78 (39%)	96 (21%)	96 (21%)	62 (31%)	78 (39%)	49 (25%)			
€1,001 to €1,500	323 (24%)	42 (21%)	110 (24%)	110 (24%)	46 (23%)	42 (21%)	52 (26%)			
€1,501 to €2,000	274 (20%)	26 (13%)	96 (21%)	96 (21%)	43 (22%)	26 (13%)	33 (17%)			
€2,001 to €3,000	256 (19%)	18 (9.0%)	95 (21%)	95 (21%)	21 (11%)	18 (9.0%)	30 (15%)			
€3,001 or more	73 (5.4%)	5 (2.5%)	19 (4.2%)	19 (4.2%)	8 (4.0%)	5 (2.5%)	11 (5.6%)			
Did not answer	65 (4.8%)	16 (8.0%)	17 (3.8%)	17 (3.8%)	8 (4.0%)	16 (8.0%)	7 (3.5%)			

Notes: *The caregiver-rated and self-report-rated measures do not necessarily correspond to the same population, as different recruitment procedures and subsample selection were employed. **Post-lyceum education, commonly referred to as Vocational Training Institutes (IEK), represents the most prevalent form of associate degree in Greece

Abbreviations: PSC-17 (Pediatric Symptoms Checklist − 17 items version), RCADS-25 (Revised Children’s Anxiety and Depression Scale − 25 items version), SNAP-IV (Swanson, Nolan, and Pelham Scale), MCHAT-R/F (Modified Checklist for Autism in Toddlers - Revised with Follow-up), CAST (Child Autism Spectrum Test), CATS-2 (Child and Adolescent Trauma Screen-2)

Table 3 summarizes the psychometric properties of each tool. Table 4 provides normative references for PSC-17 (self-report). Standardized scores and classification bands for the remaining instruments are detailed in Supplementary Tables 2 through Supplementary Table 10. Test information curves, expected total score curves, item probability functions, and item and person infit and outfit statistics for each instrument’s subscale can be consulted in Supplementary Fig. 1.1.1 to Supplementary Fig. 10.4.4. Comprehensive documentation of codes and measurement properties is also made accessible through our Open Science Framework repository (https://osf.io/crz6h/) [17].

**Table 3:** Psychometric properties of the instruments

Instrument - Rater Subscale [items]	Items	Sample (*N*)	α	ωₜ	Item Scalability (Min - Max)	Violations of monotonicity	Factor loading (Min - Max)	Residual Correlations Among Items 3rd quartile [Min; Max]	RMSEA	TLI	CFI	SRMR	Item infit mean squares (Min - Max)
**Pediatric Symptom Checklist − 17 (PSC-17) - Caregiver − 6 to 18 years**	17	1,356											
Externalizing [4, 5, 8, 10, 12, 14, 16*]*	*7*		0.89	0.89	0.41–0.57	1^a^	0.65–0.81	0.05 [-0.10; 0.09]	0.06	0.98	0.99	0.04	0.8–0.9
Internalizing *[2*,* 6*,* 9*,* 11*,* 15]*	*5*		0.89	0.89	0.5–0.6	0	0.75–0.85	0.04 [-0.06; 0.08]	0.04	0.99	1.00	0.02	0.8–0.9
Attention *[1*,* 3*,* 7*,* 13*,* 17]*	*5*		0.87	0.87	0.44–0.61	0	0.57–0.93	0.05 [-0.10; 0.14]	0.12	0.92	0.96	0.07	0.6–0.9
**Pediatric Symptom Checklist − 17 (PSC − 17) - Caregiver − 3 to 5 years**	17	200											
Externalizing *[4*,* 5*,* 8*,* 10*,* 12*,* 14*,* 16)*	*7*		0.84	0.85	0.37–0.49	0	0.58–0.9	0.12 [-0.22; 0.17]^b^	0.09	0.93	0.95	0.07	0.6–0.9
Internalizing *[2*,* 6*,* 9*,* 11*,* 15]*	*5*		0.89	0.89	0.45–0.5	0	0.7–0.85	0.09 [-0.12; 0.13]	0	1	1	0.05	0.9–1.0
Attention *[1*,*3*,*7*,*13*,*17]*	*5*		0.84	0.84	0.42–0.56	0	0.59–0.86	0.08 [-0.22; 0.16]^c^	0.05	0.98	0.98	0.06	0.7–0.9
**Pediatric Symptom Checklist (PSC) - Self-report**	17	400											
Externalizing *[4*,* 5*,* 8*,* 10*,* 12*,* 14*,* 16]*	*7*		0.85	0.85	0.3–0.42	0	0.52–0.8	0.05 [-0.07; 0.08]	0.03	0.99	0.99	0.03	0.9–1.0
Internalizing *[2*,* 6*,* 9*,* 11*,* 15]*	*5*		0.88	0.88	0.46–0.56	0	0.68–0.83	0.04 [-0.08; 0.09]	0.04	0.99	1.0	0.03	0.8–0.9
Attention *[1*,*3*,*7*,*13*,*17]*	*5*		0.83	0.84	0.36–0.51	0	0.56–0.88	0.08 [-0.1; 0.12]	0.07	0.96	0.97	0.05	0.6–1.0
**Revised Children’s Anxiety and Depression Scale - (RCADS-25) - Caregiver**	25	452											
Anxiety *[2*,* 3*,* 5*,* 6*,* 7*,* 9*,* 11*,* 12*,* 14*,* 17*,* 18*,* 20*,* 22*,* 23*,* 25]*	*15*		0.94	0.94	0.31–0.52	0	0.45–0.89	0.10 [-0.14; 0.36]^d^	0.09	0.95	0.96	0.08	0.9–1.0
Depression *[1*,* 4*,* 8*,* 10*,* 13*,* 15*,* 16*,* 19*,* 21*,* 24]*	*10*		0.94	0.94	0.41–0.59	0	0.64–0.9	0.07 [-0.14; 0.13]	0.04	0.99	0.99	0.05	0.9–1.1
**Revised Children’s Anxiety and Depression Scale - (RCADS-25) - Self-report**	25	400											
Anxiety *[2*,* 3*,* 5*,* 6*,* 7*,* 9*,* 11*,* 12*,* 14*,* 17*,* 18*,* 20*,* 22*,* 23*,* 25]*	*15*		0.97	0.97	0.5–0.64	0	0.71–0.94	0.08 [-0.16; 0.19]	0.08	0.98	0.98	0.07	0.9–1.0
Depression *[1*,* 4*,* 8*,* 10*,* 13*,* 15*,* 16*,* 19*,* 21*,* 24]*	*10*		0.97	0.97	0.59–0.69	0	0.76–0.92	0.09 [-0.15; 0.14]	0.03	0.99	0.99	0.05	0.9–1.0
**Swanson**,** Nolan and Pelham Scale (SNAP-IV) - Caregiver**	26	452											
Inattention *[1–9]*	*9*		0.95	0.95	0.57–0.69	0	0.76–0.91	-0.03 [-0.17; 0.14]	0.06	0.99	0.99	0.03	0.8–0.9
Hyperactivity *[10–14]*	*5*		0.94	0.94	0.63–0.7	0	0.79–0.91	0.07 [-0.08; 0.09]	0.02	1	1	0.02	0.8–1.0
Impulsivity *[15–18]*	*4*		0.91	0.91	0.63–0.69	0	0.8–0.91	-0.11 [-0.19; -0.09]	0.02	1	1	0.02	0.7–0.9
Oppositional *[19–26]*	*8*		0.95	0.96	0.65–0.69	0	0.8–0.91	0.1 [-0.12; 0.14]	0.08	0.98	0.98	0.05	0.9–1.0
**Modified Checklist for Autism in Toddlers - Revised (MCHAT-R/F) - Caregiver**	20	200											
Unidimensional [*1–20*]	*20*		0.92	0.93	-0.01^e^ − 0.43	0	-0.39–0.94	0.04 [-0.19; 0.28]^f^	0.02	0.99	0.99	0.08	1.0–1.3
**Child Autism Spectrum Test (CAST) - Caregiver**	*28*	200											
Social contact issues [*1*,* 2*,* 5*,* 8*,* 10*,* 11*,* 13*,* 15*,* 16*,* 17*,* 21*,* 23*,* 24*,* 27*,* 31*,* 35]*	*16*		0.88	0.88	0.11^g^ − 0.37	0	0.18^h^ − 0.99	0.03 [-0.16; 0.23]^i^	0.06	0.9	0.9	0.09	0.4^j^ − 1.2
Inflexible and repetitive behaviors [*7*,* 9*,* 18*,* 20*,* 25*,* 28*,* 29*,* 30*,* 32*,* 34*,* 36*,* 37]*	*12*		0.92	0.92	0.22^k^ − 0.61	0	0.34–0.91	0.06 [-0.12; 0.15]	0	1	1	0.06	0.9–1.0
**Child and Adolescent Trauma Screen-2 (CATS-2) - Caregiver**	20	198											
Unidimensional scale for PTSD symptoms *[16–23*,* 24 (a - d)*,* 25 (a - b)*,* 26–29*,* 30 (a - b)*,* 31–35]*			0.95	0.95	0.41–0.57	0	0.61–0.87	0.11 [-0.23; 0.3]^l^	0.09	0.96	0.96	0.07	0.9–1.1
**Child and Adolescent Trauma Screen-2 (CATS-2) - Self-report**	20	137											
Unidimensional *[16- 23*,* 24 (a - d)*,* 25 (a - b)*,* 26–29*,* 30 (a- b)*,* 31–35]*	*20*		0.97	0.97	0.4–0.64	0	0.56–0.92	0.13 [-0.26; 0.25]^m^	0.07	0.98	0.98	0.06	0.9–1.1

Abbreviations: α (Cronbach’s Alpha), ωₜ (Omega Total), CFI (Comparative Fit Index), PTSD (Post-Traumatic Stress Disorder), RMSEA (Root Mean Square Error of Approximation), SRMR (Standardized Root Mean-square Residual), TLI (Tucker-Lewis Index (TLI). Cutoffs for adequate values: α > 0.7; ωₜ > 0.7; Item scalability > 0.3; Violations of monotonicity = 0; Factor loading > 0.3; Residual correlations among items < 0.2; 3rd quartile of residual correlations < 0.37; RMSEA ≤ 0.06 (≤ 0.08 is acceptable); TLI ≥ 0.95; CFI ≥ 0.95; SRMR ≤ 0.08; Item infit mean squares ≥ 0.5 and ≤ 1.5. Notes: ^a^Item 12 presented one non-serious violation of monotonicity, with critical value estimated at 6 (values below 40 are considered acceptable) ^b^ The residuals of items 4 and 5 are correlated (-0.22). ^c^ The residuals of items 1 and 7 are correlated (-0.22). ^d^ The residuals of items 9 and 3 are correlated (0.35). ^e^ There is low item scalability for items 1 (0.2), 2 (-0.01), 3 (0.25), 5 (0.07), 12 (0.05), 14 (0.24), 17 (0.15), and 19 (0.22). ^f^ The residuals of items 5 and 12 are correlated (0.28). ^g^ Only item 15 and 17 presented scalability > 0.3. ^h^ Only item 35 presented factor loading below 0.3. ^i^The residuals of items 1 and 10 (0.225)) are correlated ^j^Only items 15 was out of expected range. ^k^There is low item scalability for items 7 (0.22) and 36 (0.27). ^l^The residuals of items 3 and 16 (0.21), 4 and 5 (0.2), 4 and 6 (0.218), 4 and 9 (0.21) and 6 and 7 (0.3), are correlated. ^m^The residuals of items 1 and 3 (-0.23), 2 and 8 (0.21), 2 and 9 (-0.25), 3 and 17 (0.245), 4 and 7 (-0.21), 5 and 8 (-0.2), 5 and 10 (-0.25), 6 and 9 (-0.2), 11 and 12 (-0.22), 11 and 14 (0.24), 11 and 19 (-0.22), 12 and 15 (-0.22), and 15 and 18 (0.211) are correlated

**Table 4:**
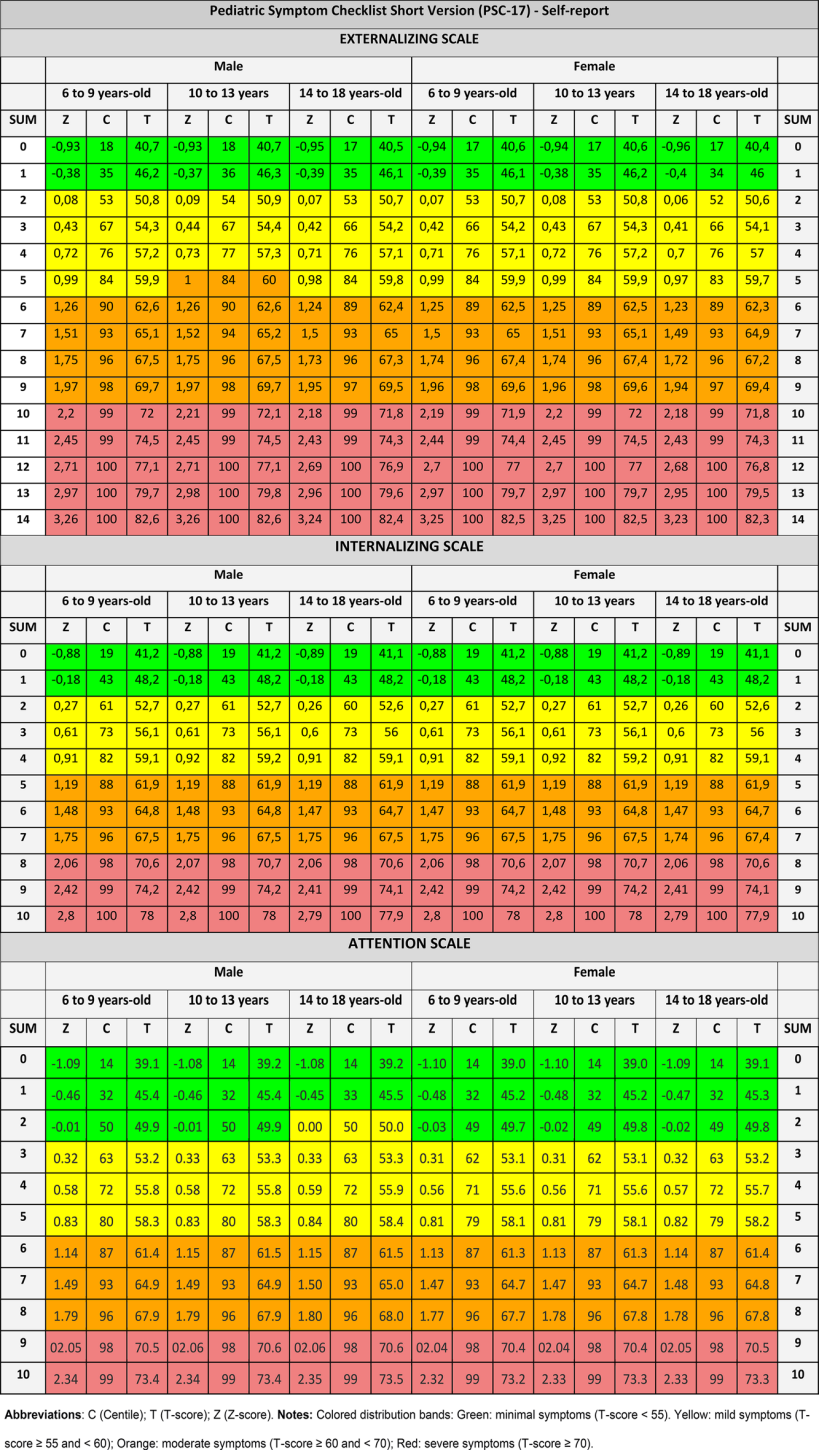
Normative references in Greece: Pediatric Symptom Checklist Short Version (PSC-17), Self-report

Internal consistency was rated as good to excellent across all subscales, with Cronbach’s alpha (α) values ranging from 0.84 to 0.97 and Omega Total (ωₜ) values from 0.85 to 0.97. Factor analysis confirmed that all scales met criteria for unidimensionality, monotonicity, local independence, and global model fit, with the exception of MCHAT-R/F (failing monotonicity), CAST - inflexible/repetitive behavior (failing monotonicity), and CAST - social contact issues (failing unidimensionality, monotonicity and model fit). Factor loadings consistently exceeded the 0.3 cutoff across tools, with a single item in CAST - social contact issues - rating 0.18. Except CAST and MCHAT-R/F, all instruments demonstrated adequate item discriminative performance and test scores.

In the CAST, both subscales failed to meet monotonicity (see Supplementary Fig. 1.1.5 and Supplementary Fig. 1.2.5 for item response functions). In the inflexible/repetitive behavior subscale, three of 15 items exhibited low scalability (< 0.3), with items 7 and 36 demonstrating weaker discrimination (see item characteristic curves in Supplementary Fig. 1.1.2). In the sociability subscale, 14 of 16 items had inadequate scalability, with item characteristic curves indicating particularly low discrimination for items 23 and 35 (Supplementary Fig. 1.2.2). Additionally, the sociability subscale failed to meet unidimensionality, as only RMSEA (0.06) fell within an acceptable range, while TLI (0.90), CFI (0.90), and SRMR (0.09) indicated inadequate global model fit.

MCHAT-R/F failed monotonicity as eight out of 20 items presented scalability values below the 0.3 threshold (the scale’s item response functions are detailed in Supplementary Fig. 4.5). Item characteristic curves indicate that some questions are unable to discriminate between respondents at both the lower and upper ends of the score spectrum (see Supplementary Fig. 4.2). For example, item 2 is overly easy to endorse, while items 5 and 12 are excessively difficult to endorse. Test information and expected score plots further indicate that the MCHAT effectively captures individuals with very low ability but fails to adequately assess those with high ability (see Supplementary Fig. 4.1).

## Discussion

This study validates and establishes local normative references for the Greek versions of six instruments: PSC-17 (caregiver- and self-report), RCADS-25 (caregiver- and self-report), CATS-2 (caregiver- and self-report), SNAP-IV (caregiver-report), MCHAT-R/F (caregiver-report), and CAST (caregiver-report). These scales demonstrated reliable and valid properties in a nationwide community sample of children, adolescents, and caregivers, with the exception of CAST and MCHAT-R/F which did not meet al.l criteria in factor analysis. To the best of our knowledge, this is the first validation of these tools in Greece apart from the prior national validation of the full-length RCADS-47 [27, 28].

Scores were categorized into symptom severity bands using PROMIS ranges for standardized metrics, which represent interpretive conventions rather than clinical cut-offs. In general, the parameters aligned with classification bands reported in Europe and North America [14, 15]. For example, a crude score of 5 on the PSC-17 internalizing subscales corresponded to a moderate symptom classification (T-score 60–69), consistent with risk cutoffs reported for samples from Spain and the United States [19, 21, 22]. On the RCADS-25 (self-report), anxiety subscale scores of 30 corresponded to a T-score of 70 (severe symptoms). This is consistent with crude-to-T-score conversion tables for boys in 3rd–4th grades and girls in 5th–6th grades from U.S. samples, yet this pattern did not hold for participants beyond the 5th grade [25, 29, 31].

Both scales assessing autism spectrum symptoms (CAST and MCHAT-R/F) did not fully meet performance criteria. This is likely due to their limited sample of 200 community-based caregivers, which is underpowered to represent individuals with more expressive clinical symptoms. Worth noting, the MCHAT-R/F is initially intended for screening children presenting developmental concerns [32]. While there is evidence of validity population-level screening, properties may differ when administered to asymptomatic samples [7]. Items were not dismissed in the scale; however, further validation in clinically representative samples is essential to confirm psychometric properties and establish clinically interpretable normative references. Accordingly, we do not endorse routine clinical application of these instruments until such validation is completed.

### Policy and practice implications

While clinical calibration is warranted before establishing score-based thresholds, these validated Greek versions offer pragmatic metrics for symptom stratification, enabling professionals to distinguish higher- from lower-symptom presentations and to monitor severity over time. This is particularly valuable for operationalising decision trees and standards of care within Greece’s national public health system, where formal gatekeeping and referral pathways remain underdeveloped. PROMIS-aligned severity bands can provide a shared clinical language to support triage, referral prioritisation, and matching of treatment intensity to need, while remaining interpretive conventions rather than diagnostic cut-offs [1, 2]. Beyond screening, these measures also support measurement-based care by tracking treatment response over the course of intervention.

To support translation into routine practice, CAMHI implementation programmes are piloting this toolkit within nationwide training initiatives for paediatricians and general practitioners focused on child and adolescent mental health assessment. Supplementary File 1 illustrates an early decision-tree card prototype for paediatric and primary-care encounters, combining universal screening (e.g., RCADS-25 and age-appropriate autism screeners such as M-CHAT-R/F or CAST) with targeted assessment for disruptive behaviour when clinically indicated (SNAP-IV). In parallel, a national initiative training psychologists and child and adolescent psychiatrists in manualised evidence-based interventions (CBT for child anxiety and adolescent depression, and behavioural parent training) is adopting RCADS-25 and SNAP-IV for outcome monitoring. Findings from these initiatives will be reported in future work and are expected to contribute to clinical validation and implementation guidance for these instruments.

### Strengths and limitations

We analyzed data from a large nationwide sample, achieving consistent psychometric properties through modern IRT approaches and generating accessible metrics that further allows for cross-instrument comparisons. However, there are also some limitations. All 1,756 caregivers were recruited from a proprietary panel of individuals willing to participate in surveys, further leading to an inclusion of 400 children and adolescents cared for by these participants. Although recruitment used census-based quotas for key sociodemographic variables to approximate the national Greek population, this strategy is not strictly probabilistic and therefore limits representativeness of our sample. Another group of 801 children/adolescents was recruited through random phone calls, a method known for low response rates and potential bias. Some tools (MCHAT-R/F, CAST, CATS-2) relied on small sample sizes (137 to 200 participants), potentially underrepresenting the full spectrum of symptoms and requiring cautious interpretation of psychometric parameters [61]. While valuable for screening symptoms and orienting referral priorities, our classification bands cannot establish sound risk stratification cutoffs, as we did not include clinical samples and comparisons with gold-standard tools [73, 74]. Although we provide age- and sex-stratified normative references, formal tests of measurement invariance and differential item functioning were not conducted in the present study. These analyses should be prioritised in future technical validation research using population-based and clinically representative samples to confirm score comparability across demographic groups.

## Conclusions

This toolkit addresses critical gaps in evidence-based resources in Greece by validating a set of widely-used tools, providing a scalable approach that can be applied to other underserved settings. Health professionals and researchers across the country are now better equipped to reliably screen and assess symptoms across the severity of symptoms in various conditions, including anxiety, ADHD, autism spectrum disorders, depression, and trauma. Implementation initiatives are currently piloting these instruments in routine care across the national health system. Future research with clinical samples and instrument comparisons are warranted to confirm normative references and establish construct and criterion validity of these tools in the country.

## Supplementary Information

Below is the link to the electronic supplementary material.

Supplementary Material 1

Supplementary Material 2

## Data Availability

Data is openly available in Open Science Framework at https://doi.org/10.17605/OSF.IO/CRZ6H, including the database and the statistical codes.
