# Supplementary material for: Psychometric properties and local normative references of PSC-17, RCADS-25, CATS-2, SNAP-IV, MCHAT-R/F, and CAST: data from a nationwide sample in Greece
Source: J Patient Rep Outcomes. 2026 Mar 11;10:62. doi: 10.1186/s41687-026-01032-1 (PMC13090465; doi:10.1186/s41687-026-01032-1)
Supplement: Supplementary file 2 — Supplementary Material 2 [file 41687_2026_1032_MOESM2_ESM.pdf]

Child & Adolescent Mental Health Initiative

Πρωτοβουλία για την Ψυχική Υγεία Παιδιών & Εφήβων

CAMHI Mental Health Screening Decision Tree

Instructions: The decision tree presented on this card has been designed for use by pediatricians and primary care providers working with children and adolescents.

For annual pediatric visits, please refer to SIDE A of the card first and make decisions based on the results derived from the **Universal Screening instruments**.

For all other pediatric visits, please begin on SIDE B of this card, administer screening instruments as indicated by current concerns, and make decisions based on the results of the **Targeted Screening instruments**.

Routine Well-Child Visit

SIDE A

Observe

Universal Screening

Scheduled mental health screening at specific timepoints; Children that you follow-up with (i.e., primary care, children with chronic conditions in specialist clinics)

Assess

Toddlers	Preschoolers	Early Childhood	Late Childhood	Adolescents
16 to 30 months	3 to 6 years	6 to 8 years	8 to 11 years	12 to 18 years
AUTISM SCREENING: M-CHAT-R*	AUTISM SCREENING: CAST*	AUTISM SCREENING: CAST* / ANXIETY SCREENING: RCADS-25	ANXIETY SCREENING: RCADS-25	ANXIETY & DEPRESSION SCREENING: RCADS-25

* These tools have not met psychometric criteria in preliminary Greek samples and should be interpreted cautiously

* M-CHAT-R/F was designed for targeted screening, and its application in universal screening remains emergent

*If **RCADS-25 item 8** is "sometimes," "often," or "always," administer the **ASQ** from **TARGETED SCREENING B**.

Communicate

Negative results or "Below Cutoff" → Discuss results and provide reassurance

Positive results or "Above Cutoff" → Discuss results and provide reassurance

Support

Negative results:

1. Provide printed ***CAMHI Wellness Resources***
2. Advise caregiver (and/or adolescent) to review ***CAMHI Short Guides*** for further information

Positive results: Proceed to **Positive Results** On **SIDE B** →

Monitor

Follow-up based on practitioner discretion

Screening based on Symptomatic Presentation

(i.e., practice, emergency department, hospital ward)

Observe

Concerns reported by child/adolescent or caregiver
or
Something concerning observed

Concerns reported about risk of harm
or
Patient is at risk of harm
(e.g., maltreatment, self-harm, etc.)

Assess

TARGETED SCREENING A:
I have a specific mental health concern

I have concerns about a child's *early development; *social communication; *restricted, repetitive behaviors:
- M-CHAT-R (16-30m) Caregiver report
- CAST (3-8y) Caregiver report

I have concerns about *excessive worries, fears, or shyness; *persistent sadness:
- RCADS-25 (6-18y) Caregiver report
- RCADS-25 (8-18y) Caregiver & self report

I have concerns about *inattention, hyperactivity, or impulsivity; *rule-breaking behavior or aggression:
- SNAP-IV (6-18y) Caregiver report

I have concerns about eating or feeding problems:
- SCOFF (≥10y) Self-report

I have concerns about alcohol or substance use:
- CRAFFT+N (12-21y) Interview or self report

*If **RCADS-25 item 8** is "sometimes," "often," or "always," administer the **ASQ** from **TARGETED SCREENING B.**

TARGETED SCREENING B:
I have a harm-related concern

I have concerns about self-injury:
- DSHI (≥12y) Self-report

I have concerns about persistent distress after traumatic experiences:
- CATS-2 (3-6y) Interview or caregiver report
- CATS-2 (7-17 y) Caregiver & self report

I have concerns about suicidal thinking or behavior:
- ASQ (≥8y) Self report

I have concerns about physical aggression or risk of violence:
- MOAS (≥7y) Interview or caregiver report

Communicate

SIDE B

MODERATE/ SEVERE Symptoms or "at or above cutoff"
→ Discuss results and provide reassurance

MINIMAL/MILD Symptoms / **NEGATIVE Results**
→ Discuss results and provide reassurance

Risk of harm
→ Discuss results and provide reassurance

Support

(Moderate/Severe)
1. Give access **CAMHI Short Guides** for further information
2 Provide printed **CAMHI Wellness Resources**

(Minimal/Mild / Negative)
1. Give access **CAMHI Short Guides** for further information
2 Provide printed **CAMHI Wellness Resources**

(Risk of harm)
1. **Activate** patient's support network and **Create** a safety/suicide prevention plan, if needed
2. Give access **CAMHI Short Guides** for further information

Monitor

Follow-up based on practitioner discretion

Follow-up in 24 to 48 hours

Guide

- Facilitate referral to a mental health professional
- Give access **CAMHI Clinical Guides** for further information

DISCLAIMER
Your judgment and clinical opinion are important!
If there is a reason to take an alternate pathway through this decision tree, that pathway should be taken.

Take child protection measures/notify social workers, police, prosecutors, etc.
(If warranted by results, referral to emergency services)

Διεθνής Πρωτοβουλία για την Υγεία
Global Health Initiative

ΙΣΝ / SNF
ΙΔΡΥΜΑ ΣΤΑΥΡΟΣ ΝΙΑΡΧΟΣ
STAVROS NIARCHOS FOUNDATION
επίκεντρο ο άνθρωπος
humanity at the core

Child Mind Institute
